# Supplementary material for: Approximate Bayesian computation with deep learning supports a third archaic introgression in Asia and Oceania
Source: Nat Commun. 2019 Jan 16;10:246. doi: 10.1038/s41467-018-08089-7 (PMC6335398; doi:10.1038/s41467-018-08089-7)
Supplement: Supplementary file 1 — Supplementary Information [file 41467_2018_8089_MOESM1_ESM.pdf]

Supplementary Information

Approximate Bayesian computation with Deep Learning supports a third archaic introgression in Asia and Oceania

Mondal et al.

Contents

Supplementary Information..1

Supplementary Methods..3

Variant Calling:..3

Filters: 4

Chimp Reference Mapping and Calling: 4

Supplementary Table 1..6

Supplementary Table 2...7

Supplementary Figure 1.. 8

Supplementary Table 3...9

Supplementary Table 4...10

Supplementary Figure 2. 11

Supplementary Note 1 11

Supplementary Figure 3a:. 15

Supplementary Figure 3b:...16

Supplementary Note 2..17

Supplementary Table 5...22

Supplementary Table 6..24

Supplementary Figure 4.. 24

Supplementary Table 7...25

Supplementary Table 8..27

Supplementary Table 9...29

Supplementary Table 10...31

Supplementary Figure 5.. 32

Supplementary Methods

Variant Calling:

FASTQ sequences were mapped using BWA mem 0.7.15 [1] on GR37 reference genome using default parameters. BWA output (SAM format) was converted to binary BAM format using SAMtools 1.3.1 [2] and sorted using picard tools 2.2.1 with SortSam using their coordinates. Then we adopted "Best Practices" recommendations from Genome analysis toolkit site [3] for every single lane BAM file. Unmapped sequences were removed using CleanSam and then duplicated reads were marked using markDuplicates both from picardtools. We realigned indels using IndelRealigner with already known indel interval file (1000 Genome Project 1st Phase Indel) [4] and recalibrated bases using BaseRecalibrator and PrintReads with dbSNP version 138 [5] using GATK 3.5. If the data came from multiple runs, the BAM files were merged using SAMtools. In case the BAM files were accessible, we directly used them for the variant calling.

After getting all the BAM files, we followed the gvcf methods for variant calling. First every BAM file was called for variants using HaplotypeCaller which produced a gVCF file for single individual. After getting all the gVCF files together, the VCF file was constructed with GenotypegVCFs using all the default parameters except max alternate alleles which was put as 20 to capture more alternate alleles as our date set is quite diverse. We used dbSNP version 138 to mark known SNPs and Indels[5].

The final VCF file was re-calibrated following GATK's Variant Quality Score Recalibration (VQSR) recommendations. VariantRecalibration from GATK was applied to calculate various statistics for both SNPs and indels using various publicly available reference datasets with known amount of false positives (dbSNP, HapMap, Omni genotype, 1000 Genomes Project; all of them were downloaded from GATK's site dated 30/08/2016). After getting the statistics, all the variants are marked using ApplyRecalibration from GATK. The commands used for VariantRecalibration step are as follows:

For SNPs:

1. HapMap 3.3: hapmap,known=false,training=true,truth=true,prior=15.0
2. Omni genotyping array 2.5 million 1000G: omni,known=false,training=true,truth=true,prior=12.0

3. 1000G phase 1 high confidence: 1000G,known=false,training=true,truth=false,prior=10.0
4. dbSNP 138: dbsnp,known=true,training=false,truth=false,prior=2.0

For Indels:

1. Mills 1000G high confidence indels: mills,known=false,training=true,truth=true,prior=12.0
2. dbSNP 138: dbsnp,known=true,training=false,truth=false,prior=2.0

Filters:

As we have used only high coverage data and D-statistics is robust for high coverage data, we adopted a minimal filter strategy (although stricter filters produced similar results, data not shown). We only retained biallelic SNPs which passed the filter tag from VQSR step using vcftools 0.1.13 [6]. We converted the VCF files to PLINK format using VCFtools and then added the Ancestral information from 1000 genome data site [4]. We removed all the SNPs which had missing information for any individual, using --geno flag in PLINK-1.07 [7] to remove lowly covered regions, which can bias the frequency estimation for populations. Although the results are similar, we removed all the transitions as ancient genomes are prone to have more transitions due to DNA degradation. The similarity is most probably due to the high coverage ancient genomes used for this study. ABC-DL method has used a different filtering approach (see below).

Chimp Reference Mapping and Calling:

To check for biases due to the use of modern human reference genome, mostly of European origin, we also remapped two persons per population on the chimpanzee reference genome (for Irula we have used data from SGDP [8]) and redone the variant calling to calculate the D-statistics and F4 ratio test. The previously created human reference mapped BAM files were split by read groups using SAMtools split. Then every split BAM files were changed to unmapped BAM files using RevertSam from picard tools and then this unmapped BAM files were converted to FASTQ files using SAMtools fastq. After that this FASTQ files were remapped on the chimpanzee reference genome (panTro4) following a similar strategy as was done on human reference genome. Although CleanSam and markDuplicates were used as human reference variant calling method, we have not used IndelRealigner and BaseRecalibration due to lack for proper reference dataset. As low coverage data can bias the variant calling towards the reference genome, which is relevant here because we have used the chimpanzee reference for D-statistics calculation (a low coverage genome would have higher number of reference allele which would be translated into higher number of

ancestral alleles), we downgraded all the BAM files towards the lowest covered genome using SAMtools view -s command. We used HaplotypeCaller to do the variant calling. Again the VariantRecalibrator step was not used as due to lack of reference data set. Then we converted the VCF file to EIGENSTRAT format and used Admixture to calculate the D-statistics. We put the reference allele as Ancestral and all the alternative alleles to Derived allele.

Supplementary Table 1: Sources of Data used in this paper.

Individual	Population	Origin of Data
HG02922	Esan in Nigeria (AFR)	1000 Genomes
HG03052	Mende in Sierra Leone (AFR)	1000 Genomes
NA19238	Yoruba in Ibadan, Nigeria (AFR)	1000 Genomes
NA19239	Yoruba in Ibadan, Nigeria (AFR)	1000 Genomes
HG00096	British in England and Scotland (EUR)	1000 Genomes
HG01500	Iberian Population in Spain (IBS)	1000 Genome
NA12891	Utah Residents (CEPH) with Northern and Western Ancestry	1000 Genomes
NA12892	Utah Residents (CEPH) with Northern and Western Ancestry	1000 Genomes
HG00419	Southern Han Chinese (ASN)	1000 Genomes
HG00759	Chinese Dai in Xishuangbanna, China (ASN)	1000 Genomes
NA18525	Han Chinese in Beijing, China (ASN)	1000 Genomes
NA18939	Japanese in Tokyo, Japan (ASN)	1000 Genomes
ERS1358131	Australian Aborigines (PAC)	SGDP
ERS1358132	Australian Aborigines (PAC)	SGDP
ERS1042142	Papuan (PAC)	SGDP
ERS1042181	Papuan (PAC)	SGDP
ERS1358076	Irula (ILA)	SGDP
ERS1358081	Irula (ILA)	SGDP
BIR-08	Birhor (IND)	Mondal et al. 2016
BIR-11	Birhor (IND)	Mondal et al. 2016
IL-01	Irula (IND)	Mondal et al. 2016
IL-04	Irula (IND)	Mondal et al. 2016
JAR-27	Jarawa (AND)	Mondal et al. 2016
JAR-32	Jarawa (AND)	Mondal et al. 2016
ONG-1	Onge (AND)	Mondal et al. 2016
ONG-12	Onge (AND)	Mondal et al. 2016
Altai	Neanderthal (NEAN)	Green et al. 2010
Vindija 33.19	Neanderthal (NEAN)	Prufer et al. 2017
Denisova	Denisova (DENI)	Meyer et al. 2012

Supplementary Table 2: Results of D-statistics analysis mapped on the human and the chimpanzee reference genome. Two Europeans (EUR: British in England and Scotland [GBR], Utah Residents (CEPH) with Northern and Western Ancestry [CEU], two East Asians (ASN: Han Chinese in Beijing, China [CHB], Japanese in Tokyo, Japan [JPT]), two Africans (AFR: Yoruba in Ibadan, Nigeria [YRI]), two Irulas (ILA), two Pacific Populations (PAC: Papuan, Australian Aborigines), two Neanderthals (NEAN: Altai and Vindija) and one Denisova (DENI). In case of human reference, the ancestral alleles are those of 1000 Genomes Project (which is a construct using human-gorilla-chimpanzee alignment) and in case of chimpanzee reference, the ancestral alleles are the reference alleles from pantro4.

**W**	**X**	**Y**	**Z**	**Human Reference D score**	**Z score**	**Chimp reference D score**	**Z score**
EUR	ASN	AFR	Ancestral	0.0008	0.333	0.0033	1.1803
EUR	IND	AFR	Ancestral	0.007	2.835	0.0319	11.5043
EUR	PAC	AFR	Ancestral	0.0337	10.651	0.0524	17.0834
AFR	EUR	NEAN	Ancestral	-0.0435	-11.453	-0.0454	-12.5242
AFR	ASN	NEAN	Ancestral	-0.0538	-11.667	-0.052	-12.4132
AFR	IND	NEAN	Ancestral	-0.0493	-12.651	-0.0551	-15.0352
AFR	PAC	NEAN	Ancestral	-0.0425	-10.322	-0.0711	-15.4772
AFR	EUR	DENI	Ancestral	-0.0051	-1.598	-0.0139	-4.6682
AFR	ASN	DENI	Ancestral	-0.0141	-4.285	-0.0202	-6.2322
AFR	IND	DENI	Ancestral	-0.02	-5.885	-0.0219	-7.0152
AFR	PAC	DENI	Ancestral	-0.0768	-16.633	-0.0731	-17.6192

Supplementary Figure 1. Considered models in the initial simulations. See the text for details.

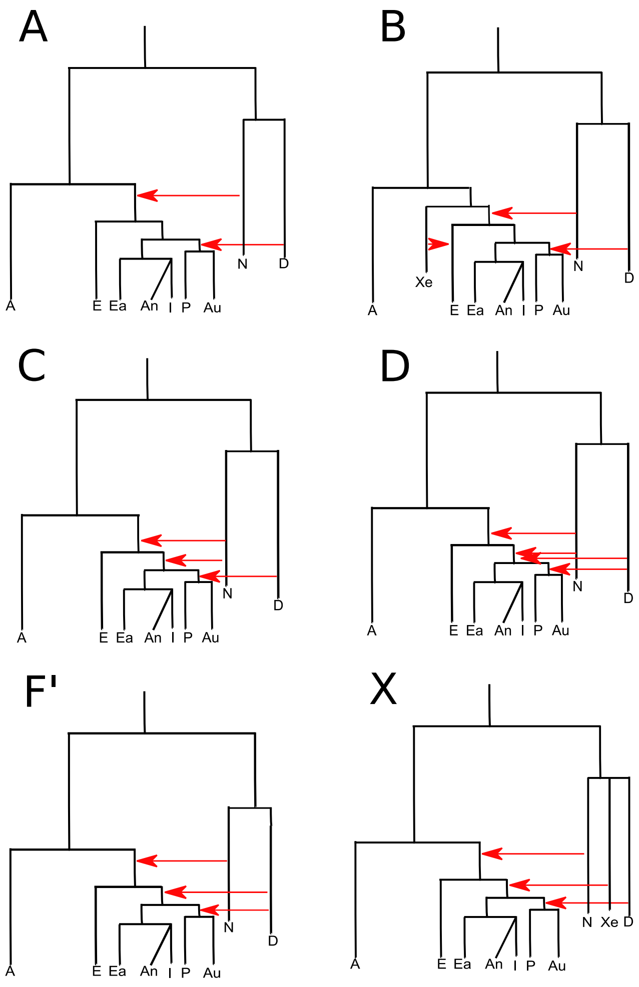

Supplementary Table 3. Parameter values for each model. Most of the values come from [14]. In case the value of the parameter is not available, we assumed a most plausible value. As D-statistics and F4 ratio are generally parameter independent, these assumptions will not affect the results.

**Model**	**Parameter**	**Value**
Common to all models	Fragment size per region	10kb
	Number of Regions	3×10^{5}
	Mutation rate	1.45×10^{-8}
	Recombination rate	1.3×10^{-8}
	Ne each population	11,273
	time Split (AND, IND, ASN)	45 kya
	time Split (PAC, ASN)	55 kya
	time Split (ASN, EUR)	60 kya
	time Split (OOA, AFR)	125 kya
	time Split (DENI, NEAN)	381 kya
	time Split (EAH, AMH)	571 kya
	time Introgression of DENI in PAC	40 kya
	time Introgression of NEAN in OOA	75 kya
Model B	time Introgression of Xe to EUR	20 kya
	time Split(Xe, OOA)	100 kya
Model C	time Introgression of NEAN to ASN	58 kya
Model D	time Introgression of NEAN to ASN	58 kya
	time Introgression of DENI to ASN	58 kya
Model F’	time Introgression of DENI to ASN	58 kya
Model X	time Split (DENI, NEAN, EEH)	381 kya
	time Introgression of EEH to ASN	58 kya

where kya=thousand years ago, AND=Andamanese, IND=Indian Tribal Populations, ASN=East Asians, PAC=Pacific/Oceania, EUR=Europeans, OOA=Out of Africa populations, DENI=Denisova, NEAN=Neanderthal, EAH=Eurasian archaic hominin, EEH=Eurasian Extinct Hominin, AMH=Anatomically Modern Human, Xe ghost population.

Supplementary Table 4. ms code for each of the considered demographic models

Model	ms code
Model A	*ms 54 300000 -I 8 8 8 8 8 8 8 4 2 -t 6.53834 -r 5.86196 10001 -es 0.0305888039 6 0.9734 -ej 0.0305888039 9 8 -ej 0.0344124044 5 3 -ej 0.0344124044 4 3 -ej 0.0420596053 6 3 -ej 0.0458832058 3 2 -es 0.0573540073 2 0.9788 -ej 0.0573540073 10 7 -ej 0.0955900121 2 1 -ej 0.291358357 8 7 -ej 0.4366551755 7 1 -seeds 100 200 300*
Model B	*ms 54 300000 -I 9 8 8 8 8 8 8 4 2 0 -t 6.53834 -r 5.86196 10001 -es 0.0152944019 2 0.7883 -ej 0.0152944019 10 9 -es 0.0305888039 6 0.9752 -ej 0.0305888039 11 8 -ej 0.0344124044 5 3 -ej 0.0344124044 4 3 -ej 0.0420596053 6 3 -ej 0.0458832058 3 2 -es 0.0573540073 2 0.9738 -ej 0.0573540073 12 7 -ej 0.0764720097 9 2 -ej 0.0917664117 2 1 -ej 0.291358357 8 7 -ej 0.4366551755 7 1 -seeds 100 200 300*
Model C	*ms 54 300000 -I 8 8 8 8 8 8 8 4 2 -t 6.53834 -r 5.86196 10001 -es 0.0305888039 6 0.9742 -ej 0.0305888039 9 8 -ej 0.0344124044 5 3 -ej 0.0344124044 4 3 -ej 0.0420596053 6 3 -es 0.0443537656 3 0.9956 -ej 0.0443537656 10 7 -ej 0.0458832058 3 2 -es 0.0573540073 2 0.9785 -ej 0.0573540073 11 7 -ej 0.0917664117 2 1 -ej 0.291358357 8 7 -ej 0.4358904554 7 1 -seeds 100 200 300*
Model D	*ms 54 300000 -I 8 8 8 8 8 8 8 4 2 -t 6.53834 -r 5.86196 10001 -es 0.030588803886002 6 0.9783 -ej 0.030588803886002 9 8 -ej 0.034412404371752 5 3 -ej 0.034412404371752 4 3 -ej 0.042059605343252 6 3 -es 0.044353765634702 3 0.9962 -ej 0.044353765634702 10 7 -es 0.045883205829003 3 0.9969 -ej 0.045883205829003 11 8 -ej 0.045883205829003 3 2 -es 0.057354007286253 2 0.9787 -ej 0.057354007286253 12 7 -ej 0.091766411658005 2 1 -ej 0.291358357014166 8 7 -ej 0.435890455375523 7 1 -seeds 100 200 300*
Model F'	mscode: *ms 54 300000 -I 8 8 8 8 8 8 8 4 2 -t 6.53834 -r 5.86196 10001 -es 0.030588803886002 6 0.9879 -ej 0.030588803886002 9 8 -ej 0.034412404371752 5 3 -ej 0.034412404371752 4 3 -ej 0.042059605343252 6 3 -es 0.044353765634702 3 0.985 -ej 0.044353765634702 10 8 -ej 0.045883205829003 3 2 -es 0.057354007286253 2 0.9783 -ej 0.057354007286253 11 7 -ej 0.091766411658005 2 1 -ej 0.291358357014166 8 7 -ej 0.435890455375523 7 1 -seeds 100 200 300*
Model X	*ms 54 300000 -I 9 8 8 8 8 8 8 4 2 0 -t 6.53834 -r 5.86196 10001 -es 0.030588803886002 6 0.9779 -ej 0.030588803886002 10 8 -ej 0.034412404371752 5 3 -ej 0.034412404371752 4 3 -ej 0.042059605343252 6 3 -es 0.044353765634702 3 0.984 -ej 0.044353765634702 11 9 -ej 0.045883205829003 3 2 -es 0.057354007286253 2 0.9789 -ej 0.057354007286253 12 7 -ej 0.091766411658005 2 1 -ej 0.291358357014166 9 7 -ej 0.291358357014166 8 7 -ej 0.435890455375523 7 1 -seeds 100 200 300*

Supplementary Figure 2. Flowchart example to calculate Introgression amount

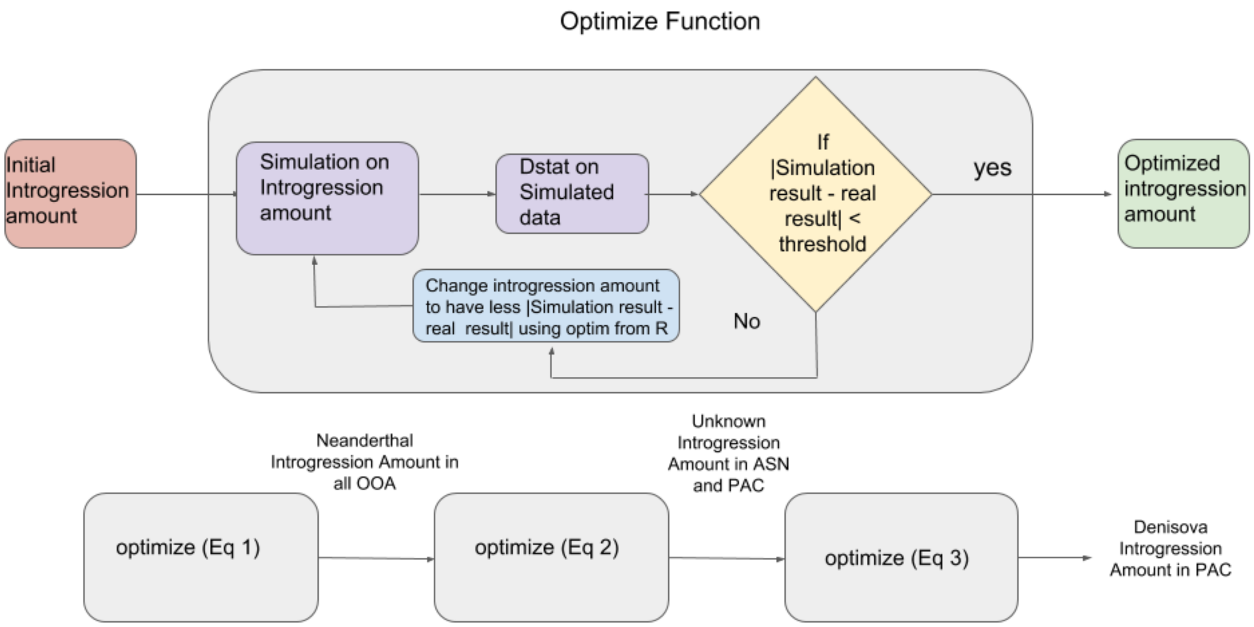

Supplementary Note 1: D-statistics and F4 ratio test for estimating introgression proportions in simple demographic scenarios.

We run all the simulations with ms >(see Supplementary Table 4). We used five D-statistics results to train our dataset for different simulation models:

1) D-statistics (EUR, AFR, NEAN, Ancestral) = 0.0457 (Equation 1).
2) D-statistics (ASN, AFR, NEAN, Ancestral) = 0.0557 (Equation 2).
3) D-statistics (PAC, AFR, DENI, Ancestral) = 0.0753 (Equation 3).
4) D-statistics (ASN, EUR, NEAN, Ancestral) = 0.0126 (Equation 4).
5) D-statistics (ASN, EUR, DENI, Ancestral) = 0.0096 (Equation 5).

Model A:

Under this model, there is an introgression of Neanderthal to all OOA population and one of Denisova in the Pacific. We first estimated the Neanderthal introgression amount in all OOA populations using Equation 1 and then Equation 3 was used to calculate the introgression amount of Denisova in Pacific populations.

Model B:

We first solved the Equation 2 to find the Neanderthal introgression for all OOA populations. Then we estimated the amount of admixture needed from non-introgressed population to European using Equation 1 and at the end Equation 3 was evaluated to find the amount of Denisova introgression in Pacific population.

Model C:

This model assumes two Neanderthal introgressions and one from Denisovans. In it, we first solved the Neanderthal introgression amount for all OOA populations using Equation 1. Then we estimated the second Neanderthal introgression amount using Equation 2 and in the end the amount of Denisova introgression was calculated using Equation 3.

Model D:

In this model, there are two Neanderthal and two Denisova introgressions. Again, Equation 1 was used to calculate Neanderthal introgression for all OOA populations. Then Equation 4 and 5 was solved together which gave the amount of Neanderthal introgression and Denisova introgression for Asian populations. After that we estimated Denisova introgression amount for Pacific populations using Equation 3.

Model F':

This model assume one introgression from Neanderthal to all OOA, one introgression from Denisova to all Asia and Pacific population and then again another Denisova to Pacific population only. The Neanderthal introgression amount was calculated using Equation 1. Then using Equation 2, we calculated the introgression from Denisova for Asian populations and then using Equation 3 we estimated the Denisova introgression in Pacific populations.

Model X:

We have used the Equation 1 first to calculate the amount of Neanderthal introgression for all OOA populations. Then we used Equation 2 to calculate the amount of introgression from unknown EEH population to Asia and Pacific population and in the end we estimated Denisova introgression to Pacific population using Equation 2.

After estimation of above mentioned parameters for every model, we then re-calculated some of D-

statistics and F4 ratio test for those models to compare which model is the best explaining the empirical results (Supplementary Figure 1).

After optimization of the introgression amount, we ran ms code for the final time with 10 kb regions and 3×10^5 replicates, which is converted to 3×10^9 base pair region per individual. Then we converted ms output to eigenstrat format and run qpDstat on the simulated data with 859 blocks for block jack knife. We then compared real D-statistics and F4 ratio results (value, standard error and 555 blocks for Jackknife) with the simulated D-statistics and F4 ratio results (value, standard error and 859 blocks for Jackknife) using a simple t-tests to calculate the p value (the number of blocks were used to calculate the degrees of freedom).

We simulated above mentioned scenarios (3×10^5 replicates of 10kb regions) to calculate D-statistics and F4 ratio tests. We reached the conclusion from the results with these statistics that the best-fitted model was given by the introgression in all Asian populations from an unknown EEH population (Model X), though the Model of double introgression from Denisova and Neanderthal (Model D) came close to explain the results (Supplementary Figure 3).

The no introgression model (Model A) failed to explain the higher Neanderthal and Denisova ancestry in Asian populations compared to Europeans using D-statistics (Supplementary Figure 3a). It is interesting to note that the increase of Neanderthal ancestry in Asian populations compared to Europeans is >20% when the Ancestral was used as the out-group, but this increase diminished when the Denisova was used as the out-group in the F4 ratio test (Supplementary Figure 03b). A similar result was observed with D-statistics (data not shown). Our results also reject the Model B (dilution of Neanderthal ancestry in Europeans) and Model C (two introgressions for Asians from Neanderthals) since F4 ratio test should be independent of the out-group in those models and should show exactly the same amount. Model F' shows an increase in the amount of Denisova ancestry much higher in D-statistics but that reduces the Neanderthal ancestry detected by F4 ratio tests; the model, thus, can be rejected (Supplementary Figure 3).

This phenomenon is much better explained by the scenario of double introgression from both Neanderthal and Denisova (Model D) and the introgression from an unknown EEH population (Model X). Introgression from any EEH population, which is an out-group or in trichotomy with Neanderthal and Denisova separation (but not an out-group of modern human and Neanderthal-

Denisova lineage), would increase the D-statistics values of Neanderthal and Denisova, as this EEH population shared an ancestry with them. The increase would be more or less similar as this population would share derived alleles present both in Neanderthal and Denisova due to incomplete lineage sorting. If the introgression happened either from Neanderthal or Denisova, the increase in D-statistics would not be uniform (Model C, Model E and Model F'). In contrast, F4 ratio test detects a differential Neanderthal ancestry when using different outgroups (Supplementary Figure 03b). Interestingly under Model X, Asian populations should have a dearth of African alleles like Andamanese (data not shown); we detected this lack of African ancestry in case of Indian and Andamanese populations (as well as in a Tibeto-Burman population) but failed to detect it in case of East Asian populations (Mondal et al. 2016). These results were reproduced in an independent dataset using Chimp reference (see above).

The alternative scenario of two independent introgressions with the similar amount from Neanderthal and Denisova in Asian populations (Model D) can also explain the results of D-statistics and F4 ratio tests and cannot be ruled out on these basis only. Although this is highly unlikely in practicality, as the time span between Asian populations had separated from the Europeans and their separation from each other is small. To solve this dilemma, we used ABC-DL method which is much better suited to delimit this kind of situation (see later).

In the case of Pacific populations, although they are known to have introgression from Denisova, we found that Papuan has much more Neanderthal ancestry than other OOA populations when using D-statistics (Table 1). But this higher Neanderthal ancestry in Pacific populations can be explained by the high Denisova introgression in these populations due to the shared ancestry between them.

Supplementary Figure 3a: Neanderthal and Denisova introgression amount of Asian populations compared to Europeans calculated by D-statisticss for real and different simulation models (ModelA-ModelX). D-statistics (European, X, Neanderthal, Ancestral) is in red and D-statistics (European, X, Denisova, Ancestral) is in Black. X is either East Asian, Indian Tribal or Andamanese populations. The bar represents the standard error. P values were calculated by t-test between the model and the real results (values lower than .05 were marked with red color). Standard error was calculated using Jackknife on 555 blocks for real data and on 859 blocks for simulated data.

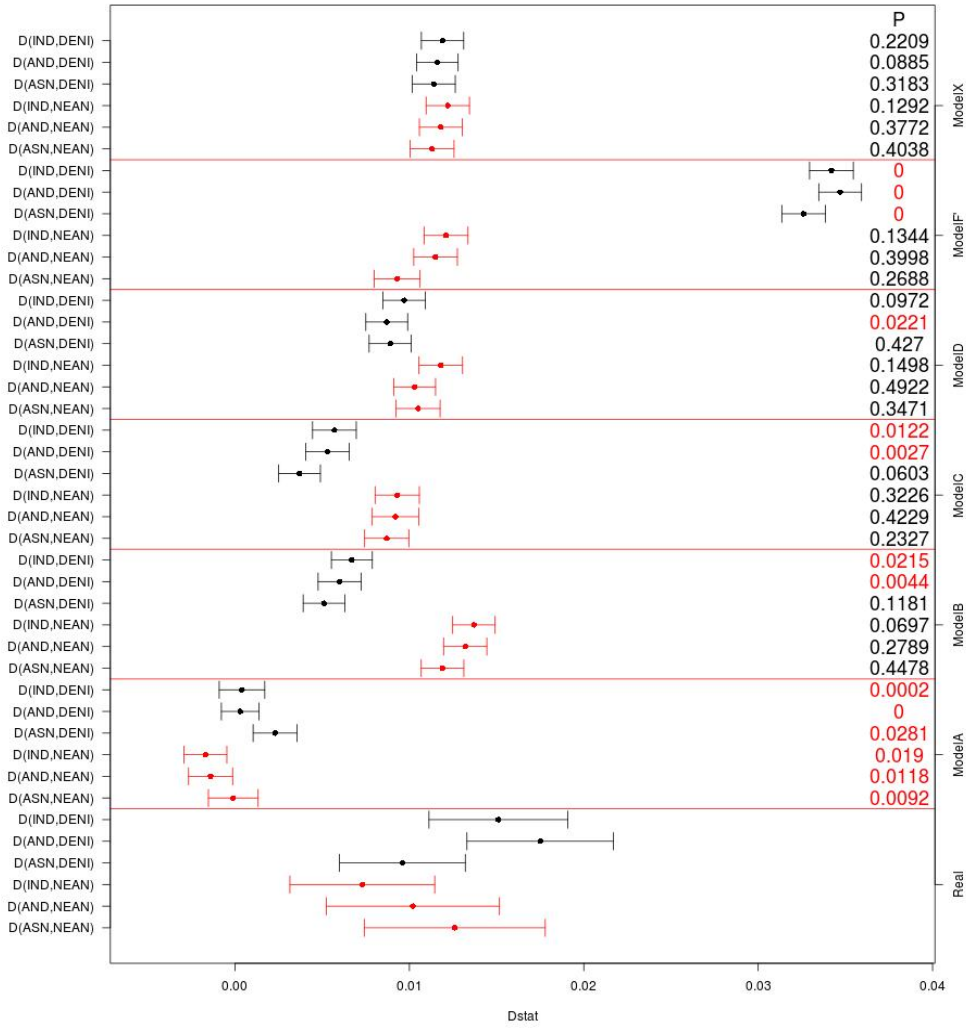

Supplementary Figure 3b: Neanderthal introgression amount of Asian populations compared to Europeans calculated by F4 ratio test for real and different models (ModelA-ModelX). F4 ratio (Denisova, Altai Neanderthal; Africa, X: Denisova, Altai Neanderthal; Africa, Vindija Neanderthal). X is either East Asian, Indian Tribal or Andamanese populations. The bar represents the standard error. P values were calculated by t-test between the model and the real results (values lower than .05 were marked with red color). Standard error was calculated using Jackknife on 555 blocks for real data and on 859 blocks for simulated data.

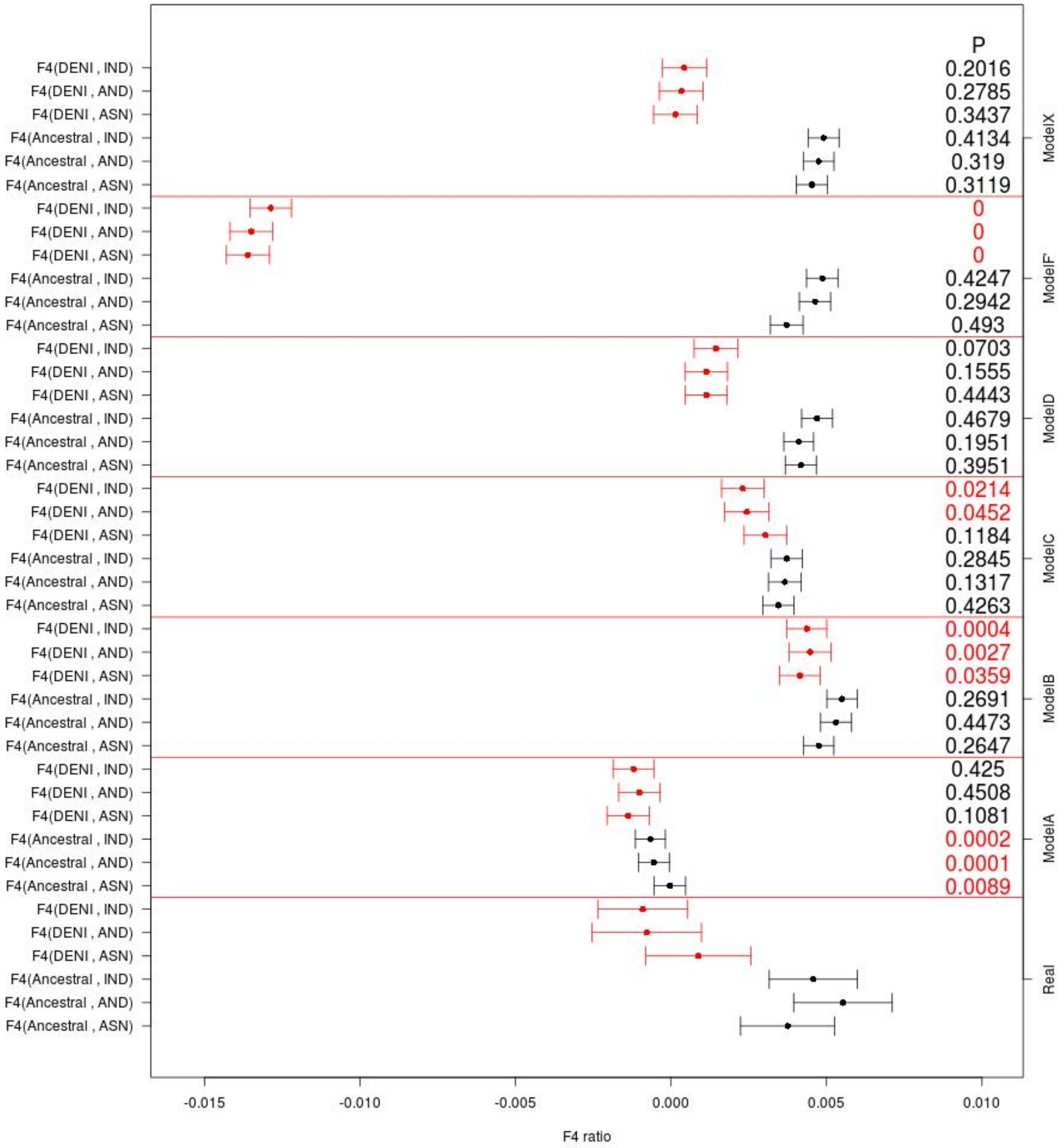

Supplementary Note 2: Approximate Bayesian Computation (ABC) coupled to a Deep Learning (DL) approach (ABC-DL)

Data preprocessing

We considered the individuals for ABC-DL analyses: *Altai, BIR-08, BIR-11, Denisova, ERS1358131, ERS1358132, ERS1042142, ERS1042181, HG00096, HG00419, HG00759, HG01500, HG02922, HG03052, IL-01, IL-04, JAR-27, JAR-32, ONG-1, ONG-12, NA12891, NA12892, NA18525, NA18939, NA19238 and NA19239* (Supplementary Table 1).

Data cleaning comprised:

1. Masking genomic regions containing Ensembl genes ± 20kb.
2. Masking CgP islands defined in [1]>
3. Retrieving genomic regions of at least 10kb and separated by at least 100kb. Within each genomic fragment we concatenate genomic fragments that were at <=5 Kb from each other and that were neither in genes nor not in CgP islands.

After data cleaning, the total number of considered regions was 9,643, comprising 651 Mb.

Demographic Models

The considered demographic models are depicted in Figure 1. All models considered a tree topology shaped by the OOA, a diaspora and further admixture with other archaic populations. The main interest is in identifying the archaic sources of archaic introgression in Asian and Australian/Pacific populations, thus the evolutionary structure of the populations of modern humans is taken as the same in all models and it is not tested. Supplementary Table 5 shows the prior distributions for the different parameters considered in at least one of the compared models.

For the parameters comprising effective population sizes, we considered a uniform distribution of priors with a broad interval of possible values. In the case of the time of splits, we considered Uniform priors distributed around previously estimated values in the literature with broad ranges. In the case of the time of split between Neanderthals and Denisovans, we defined a broad range focusing on the values reported by [1]>, which used a comparable mutation rate as the considered in this study.

Deep Learning

Artificial Neural Networks (ANNs) are a biologically-inspired programming machine learning paradigm which enables a computer to learn from observational data. ANNs are able to model complex non-linear input–output mappings and are particularly suited for solving universal

problems to many domains of science, business and government [1]>. Deep learning (DL) composes of simple but non-linear modules that each transforms the representation at one level (starting with the raw input) into a representation at a higher, slightly more abstract level [13]. It has been recently suggested as an approach for estimating demographic parameters in complex demographic scenarios [14]. Each ANN is defined by a large number of hyperparameters, including the neural network topology (number of layers, number of neurons by layer, connections between neurons within and between layers), the type of activation function of each neuron/layer (and associated parameters), the learning algorithm for estimating the weights of the connections between neurons (and parameters that define the ascertained strategy) and algorithms for alleviating the problem of overfitting the ANN with the training data such as early stop training, dropout [13] and noise injection [15], among others.

ABC-DL Implementation for Model inference

Given the exponential nature of the multivariate joined SFS, considering the nine populations -each population sampled at two chromosomes- for model comparison comprises 19,681 (3^9-2) cells as input for the DL. Such a large number of input features can introduce problems of overfitting and it is computationally intensive. In order to reduce the number of observed cells allowing to the ANN while keeping the inner topology of the models, we excluded from the analyses the SFS cells corresponding to the Indian and Papuan populations, leading to 3^7 -2 = 2,185 SFS cells. Furthermore, in order to reduce the variance and magnitude among different SFS cells, we standardized each SFS cell among the different simulations. We generated a supervised four layer feedforward DL network for inferring the demographic model using the 2,185 SFS cells. Input layer (top) corresponds to the SFS computed using seven populations: A (African), E (Europeans), EA (East Asian), AN (Andamanese), AU (Australian Aborigines), N (Neanderthal) and D (Denisova). Output layer (bottom) corresponds to one of the eight proposed models. Hidden layers consider a bias unit and 200 neurons with Elliot activation functions [1]>with a modified slope s at 0.1 to enhance linearity:

$$f(x) = \frac{sx}{2(1 + |sx|)} + 0.5$$

Training of the DL applied resilient propagation with a dropout rate of 0.5, a RPROP algorithm for adaptive learning [1]> and noise injection as described previously. The proposed DL network is

implemented using Encog3.4 [17] after extending the framework to include noise injection.

ABC Implementation for Model identification

For DL training, we generated 15,000 simulations from each of the eight considered models, comprising a total of 120,000 simulations. We run 10 independent supervised DL networks with these simulations. Noise injection was introduced using the observed SFS computed from *HG02922, NA12891, HG00419, ONG-1, ERS1358131, Altai* and *Denisova*. Each DL network was trained to identify the model used to generate each simulation by means of a SoftMax layer. It was run until error decreased up to 0.01 or it has run for more than 10,000 iterations. The output of each DL prediction for a given simulation was combined to produce a unique output in bagging [1]>, and the combined prediction was used for conducting a local linear regression ABC approach. We generated additional 100,000 simulations of each demographic model. For each simulation, we use the DLs to predict the model that generated it. In parallel, we estimated the model probabilities at each DL network with observed data not used for training or replication dataset (with the exception of Neanderthal Altai and Denisovan): *NA19239, NA12892, NA18939, ONG-12* and *ERS1358132*.

In order to evaluate the power of ABC for distinguishing between these eight independent models, we ran a leave one-out cross-validation algorithm using 100 randomly sampled simulations from each model in the replication dataset as "observed data", and using the remaining 7 x 14,900 simulations for the ABC. We considered the classical ABC rejection algorithm, retaining the closest 1,000 simulations to the observed data.

We next applied the ABC approach to the observed data using local linear regression to weight the accepted simulations. Supplementary Figure 4 shows the posterior distribution obtained for each of the eight considered models. ABC model ascertainment supports the presence of an additional archaic Ghost population introgressing in Asia besides Neanderthal and Denisovan (models E, F, G and H). The model that considers the Ghost population as an admixture of the Neanderthal and Denisovan population (Model H) was displayed to be getting the highest support, being only 1.2 times more likely than the next model with the largest posterior probability (Model F).

ABC-DL Implementation for Parameter identification

We next applied the ABC-DL implementation to model H. From the SFS, we generated the multidimensional unfolded site frequency spectrum -SFS- between all possible triplets of populations, thus reducing the total number of cells of the SFS considering all populations at once.
For each parameter of model H we trained a DL using 10,000 simulations. The DL of each parameter was generated using a four-layer feedforward network. Input layer consisted on all the SFS computed between all possible triplets of populations. Each cell was standardized using the observed values in the 10,000 simulations. The output consisted on the scaled value of the parameter of interest, ranging between 0 and 1. We considered two hidden layers of 100 neurons each, using Elliot activation functions as described previously, and a bias neuron.
We applied noise injection approach as previously described for minimizing overfitting and increasing parameter inference robustness.
Next, we generated 100,000 additional simulations, computed the SFS and applied the trained DL to get the SS to be used at the ABC. For each parameter, we first analyzed the power of the DL for predicting the value used to generate a simulation given the observed SFS between triplets of populations. Supplementary table 07 shows the estimated correlation between the predicted value by the DL and the used value to generate the simulation.
Next, we applied the ABC-DL approach to the observed data (Supplementary Table 8 and 9 and Figure 3), retaining the best 1,000 simulations out of the 100,000.
Given that the time of admixture between Denisovans and Neanderthals to create the ghost population overlaps with the time of split between Denisovans and Neanderthals, we wondered if a similar trend of overlap could be observed in other models including the archaic population. Therefore, we conducted the ABC-DL approach with model E, F and G (Supplementary Table 10).
We observe that the ABC-DL provides posterior distributions of the split of the proposed archaic ghost population tend to overlap with the time of split between Neanderthal and Denisovan.

Supplementary Table 5. Prior distributions of the considered models

Parameter	Distribution	A	B	C	D	E	F	G	H
**migration_African_to_European**	*U(0.0,5.0E-4)*	X	X	X	X	X	X	X	X
**migration_European_to_African**	*U(0.0,5.0E-4)*	X	X	X	X	X	X	X	X
**migration_African_to_EAsia**	*U(0.0,5.0E-4)*	X	X	X	X	X	X	X	X
**migration_EAsia_to_African**	*U(0.0,5.0E-4)*	X	X	X	X	X	X	X	X
**migration_European_to_EAsia**	*U(0.0,5.0E-4)*	X	X	X	X	X	X	X	X
**migration_EAsia_to_European**	*U(0.0,5.0E-4)*	X	X	X	X	X	X	X	X
**migration_Papuan_to_Australian**	*U(0.0,5.0E-4)*	X	X	X	X	X	X	X	X
**migration_Australian_to_Papuan**	*U(0.0,5.0E-4)*	X	X	X	X	X	X	X	X
**NeAfrican**	*U(10000.0,100000.0)*	X	X	X	X	X	X	X	X
**NeEuropean**	*U(1000.0,20000.0)*	X	X	X	X	X	X	X	X
**NeEastAsian**	*U(1000.0,20000.0)*	X	X	X	X	X	X	X	X
**NeAndamanese**	*U(1000.0,20000.0)*	X	X	X	X	X	X	X	X
**NeIndian**	*U(1000.0,20000.0)*	X	X	X	X	X	X	X	X
**NePapuan**	*U(1000.0,20000.0)*	X	X	X	X	X	X	X	X
**NeAustralian**	*U(1000.0,20000.0)*	X	X	X	X	X	X	X	X
**NeNeanderthal**	*U(1000.0,20000.0)*	X	X	X	X	X	X	X	X
**NeDenisovan**	*U(1000.0,20000.0)*	X	X	X	X	X	X	X	X
**NeErectus**	*U(1000.0,20000.0)*	X	X	X	X	X	X	X	X
**tSplitPapua_AA***	*U(14.5,43.5)*	X	X	X	X	X	X	X	X
**NePapua_AA**	*U(1000.0,20000.0)*	X	X	X	X	X	X	X	X
**tsplitAndamese_India***	*U(14.5,43.5)*	X	X	X	X	X	X	X	X
**NeEAsia_India**	*U(1000.0,20000.0)*	X	X	X	X	X	X	X	X
**tSplitAsia_Pacific***	*U(43.5,52.2)*	X	X	X	X	X	X	X	X
**NeAsia_Pacific**	*U(1000.0,20000.0)*	X	X	X	X	X	X	X	X
**tSplitEuropean_Asia_Pacific**	*U(1800.0,2500.0)*	X	X	X	X	X	X	X	X
**NeOutOfAfrica**	*U(1000.0,20000.0)*	X	X	X	X	X	X	X	X
**tSplitEurasia_Africa***	*U(tSplitEuropean_Asia_Pacific,174)*	X	X	X	X	X	X	X	X
**NeHumans**	*U(1000.0,40000.0)*	X	X	X	X	X	X	X	X
**NeBottleneckEurasia**	*U(1000.0,5000.0)*	X	X	X	X	X	X	X	X
**NeArchaics**	*U(1000.0,80000.0)*	X	X	X	X	X	X	X	X
**tSplitNeanderthal_Denisova***	*U(300,400)*	X	X	X	X	X	X	X	X
**NeHominin**	*U(1000.0,40000.0)*	X	X	X	X	X	X	X	X
**tHominin***	*U(450,600)*	X	X	X	X	X	X	X	X
**tIntrogressionDenisovan_Pacific***	*U(tSplitPapua_AA,tSplitAsia_Pacific)*	X	X	X	X	X	X	X	X
**IntrogressionDenisovan_Pacific**	*U(1.0E-9,0.05)*	X	X	X	X	X	X	X	X
**tIntrogressionErectus_Denisovan***	*U(41,70)*	X	X	X	X	X	X	X	X
**IntrogressionErectus_Denisovan**	*U(1.0E-9,0.05)*	X	X	X	X	X	X	X	X
**NeErectus_Hominin**	*U(5000.0,100000.0)*	X	X	X	X	X	X	X	X
**tHominin_Erectus***	*U(1000,2000)*	X	X	X	X	X	X	X	X

**tIntrogressionNeanderthal_ModernHumanOutOfAfrica***	*U(tSplitEuropean_Asia_Pacific,tSplitEurasia_Africa)*	*X*	*X*	*X*	*X*				
**IntrogressionNeanderthal_Eurasia**	*U(1.0E-9,0.05)*	*X*	*X*	*X*	*X*				
**NeGhostEuropean**	*U(2000.0,40000.0)*		*X*						
**NeBeforeSplitEurope_Asia_Pacific**	*U(1000.0,NeEastAsian)*		*X*						
**tSplitGhostEuropean_OtherModernHumans***	*U(tSplitEuropean_Asia_Pacific,tSplitEurasia_Africa)*		*X*						
**tIntrogressionGhostEurope_Europe***	*U(10,tSplitEuropean_Asia_Pacific)*		*X*						
**IntrogressionGhostEurope_Europe**	*U(1.0E-9,0.05)*			*X*					
**IntrogressionNeanderthal_Eurasia**	*U(1.0E-9,0.05)*			*X*					
**tIntrogressionNeanderthal_Asia***	*U(tSplitAsia_Pacific,tSplitEuropean_Asia_Pacific)*			*X*	*X*				
**IntrogressionNeanderthal_Asia**	*U(1.0E-9,0.05)*				*X*				
**tIntrogressionDenisovan_Asia***	*U(tSplitAsia_Pacific,tSplitEuropean_Asia_Pacific)*				*X*				
**IntrogressionDenisovan_Asia**	*U(1.0E-9,0.05)*				*X*				
**NeUnknown**	*U(1000.0,20000.0)*					*X*	*X*	*X*	*X*
**tIntrogressionAsia_Pacific***	*U(tSplitAsia_Pacific,tSplitEuropean_Asia_Pacific)*					*X*	*X*	*X*	*X*
**IntrogressionUnknown_Asia_Pacific**	*U(0.5,0.51)*					*X*	*X*	*X*	*X*
**tIntrogressionNeanderthal_ModernHumanOutOfAfrica***	*U(tSplitEuropean_Asia_Pacific,tSplitEurasia_Africa)*					*X*	*X*	*X*	*X*
**IntrogressionNeanderthal_Eurasia**	*U(1.0E-9,0.05)*					*X*	*X*	*X*	*X*
**tSplitNeanderthal_Unknown***	*U(200,tSplitNeanderthal_Denisova)*					*X*			
**tSplitDenisovan_Unknown***	*U(200,tSplitNeanderthal_Denisova)*						*X*		
**tSplitArchaic_Unknown***	*U(tSplitNeanderthal_Denisova,tHominin)*							*X*	
**IntrogressionNeanderthal_Eurasia**	*U(1.0E-9,0.05)*								*X*
**tAdmixtureNeanderthal_Denisovan_to_Unknown***	*U(tSplitEuropean_Asia_Pacific,75.4)*								*X*

* kya assuming a generation time of 29 years [1]>

Supplementary Table 6. Confusion matrix for the eight considered models using 100 samples from each model. For each simulated model (row) we performed a classical ABC rejection algorithm and identified the model (column) with the highest posterior probability.

Model	A	B	C	D	E	F	G	H	P(Msim | Mabc)
**A**	**62**	0	0	0	2	3	33	0	0.58
**B**	6	**82**	1	6	0	0	5	0	0.86
**C**	0	5	**87**	3	1	0	0	4	0.94
**D**	5	7	1	**54**	5	8	8	12	0.58
**E**	6	0	1	2	**57**	14	9	11	0.57
**F**	4	0	0	7	6	**68**	13	2	0.66
**G**	20	1	0	1	1	2	**74**	1	0.50
**H**	3	0	2	20	27	8	4	**36**	0.54

Supplementary Figure 4. Posterior probabilities of the models. They have been estimated by means of classical rejection ABC algorithm at the *NA19239, NA12892, NA18939, ONG-12, AUS2, Altai* and *Denisovan* using 100,000 simulations of each model and the combined classification output from 10 DL networks.

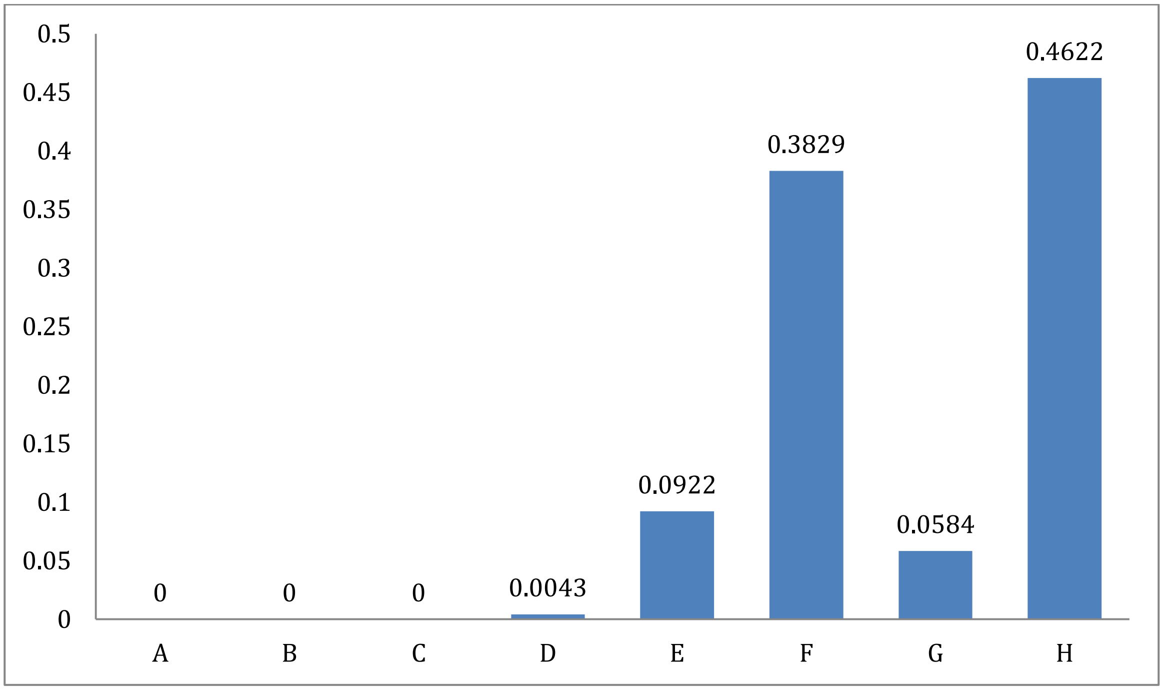

Supplementary Table 7. Linear correlation between the parameter values used for the simulation and the predicted parameter value by the DL using the SFS between triplets of populations in the replication dataset of 100,000 simulations. In bold, values <=0.1

**Parameter**	**correlation**
migration_African_to_European	0.82
migration_European_to_African	0.74
migration_African_to_EAsia	0.86
migration_EAsia_to_African	**-8.13E-05**
migration_European_to_EAsia	0.80
migration_EAsia_to_European	0.88
migration_Papuan_to_Australian	0.44
migration_Australian_to_Papuan	0.28
NeAfrican	0.78
NeEuropean	0.91
NeEastAsian	0.91
NeAndamanese	0.92
NeIndian	0.93
NePapuan	0.92
NeAustralian	0.89
NeNeanderthal	0.95
NeDenisovan	0.94
NeErectus	**-0.0005**
tSplitPapua_AA	0.52
NePapua_AA	0.63
tsplitAndamese_India	0.81
NeEAsia_India	0.82
tSplitAsia_Pacific	0.32
NeAsia_Pacific	0.82

tSplitEuropean_Asia_Pacific	0.51
NeOutOfAfrica	0.74
tSplitEurasia_Africa	0.84
NeHumans	0.55
NeBottleneckEurasia	0.25
NeArchaics	0.90
tSplitNeanderthal_Denisova	0.74
NeHominin	0.94
tHominin	0.86
tIntrogressionDenisovan_Pacific	0.53
IntrogressionDenisovan_Pacific	0.94
tIntrogressionErectus_Denisovan	0.43
IntrogressionErectus_Denisovan	0.85
NeErectus_Hominin	0.83
tHominin_Erectus	0.40
NeUnknown	**0.01**
tIntrogressionAsia_Pacific	0.39
IntrogressionUnknown_Asia_Pacific	0.87
tIntrogressionNeanderthal_ModernHumanOutOfAfrica	0.68
IntrogressionNeanderthal_Eurasia	0.90
*AdmixtureDenisovanUnknown*NeDenisovan*	0.71
tAdmixtureNeanderthal_Denisovan_to_Unknown	0.49

Supplementary Table 8. Mean and 95% Credible Interval (CI) of the posterior distributions of the demographic parameters of model H.

Parameter	mean	2.5% CI	97.5% CI
migration_African_to_European	0.00005	0.00000	0.00018
migration_European_to_African	0.00007	0.00000	0.00018
migration_African_to_EAsia	0.00021	0.00005	0.00039
migration_EAsia_to_African	0.00024	0.00001	0.00049
migration_European_to_EAsia	0.00036	0.00018	0.00049
migration_EAsia_to_European	0.00003	0.00000	0.00009
migration_Papuan_to_Australian	0.00026	0.00002	0.00049
migration_Australian_to_Papuan	0.00018	0.00001	0.00048
NeAfrican	23291.15	11190.96	45588.44
NeEuropean	8174.85	5012.68	13580.43
NeEastAsian	8630.78	5311.79	14067.76
NeAndamanese	2869.95	1293.32	4775.42
NeIndian	9782.06	6184.08	14532.16
NePapuan	5115.38	3278.30	7938.23
NeAustralian	11837.07	6681.24	18573.15
NeNeanderthal	4230.31	1257.00	6803.71
NeDenisovan	3631.35	1217.03	5976.19
NeErectus	10508.45	1468.96	19416.30
*tSplitPapua_AA**	31.88	16.68	43.12
NePapua_AA	10553.00	2617.73	19020.94
*tsplitAndamese_India**	39.54	31.67	43.37
NeEAsia_India	15682.21	7819.05	19898.89
*tSplitAsia_Pacific**	46.95	43.67	51.81
NeAsia_Pacific	15289.21	8173.67	19842.44
*tSplitEuropean_Asia_Pacific**	57.85	52.30	69.63

NeOutOfAfrica	2796.88	1091.66	5994.99
*tSplitEurasia_Africa**	121.38	78.51	166.98
NeHumans	28414.16	10934.23	39402.11
NeBottleneckEurasia	3507.75	1263.40	4939.78
NeArchaics	15091.50	6502.20	24674.82
*tSplitNeanderthal_Denisova**	314.07	300.31	343.23
NeHominin	23156.59	14956.49	33115.30
*tHominin**	558.22	503.66	596.01
*tIntrogressionDenisovan_Pacific**	43.10	29.38	50.29
IntrogressionDenisovan_Pacific	0.016	0.004	0.025
*tIntrogressionErectus_Denisovan**	77.90	44.63	98.51
IntrogressionErectus_Denisovan	0.013	0.003	0.029
NeErectus_Hominin	55395.71	19859.65	93142.14
*tHominin_Erectus**	1492.86	1021.66	1973.87
NeUnknown	10533.09	1441.02	19455.94
*tIntrogressionAsia_Pacific**	51.03	45.01	58.04
IntrogressionUnknown_Asia_Pacific	0.026	0.007	0.047
*tIntrogressionNeanderthal_ModernHumanOutOfAfrica**	69.47	56.20	88.65
IntrogressionNeanderthal_Eurasia	0.013	0.002	0.026
AdmixtureDenisovanUnknown	0.511	0.079	0.792
*tAdmixtureNeanderthal_Denisovan_to_Unknown**	304.41	211.20	375.21

* KYA assuming a generation time of 29 years.

Supplementary Table 9. Mean and 95% Credible Interval (CI) of the posterior distributions of the demographic parameters of model F.

Parameter	mean	2.5% CI	97.5% CI
migration_African_to_European	5.58E-05	1.84E-06	0.00016
migration_European_to_African	4.95E-05	1.41E-06	0.00014
migration_African_to_EAsia	0.00016	2.24E-05	0.00034
migration_EAsia_to_African	0.00025	1.48E-05	0.00049
migration_European_to_EAsia	0.00042	0.00028	0.00049
migration_EAsia_to_European	3.08E-05	1.17E-06	9.64E-05
migration_Papuan_to_Australian	0.000235	1.79E-05	0.000474528
migration_Australian_to_Papuan	0.00021	9.40E-06	0.000476128
NeAfrican	30504.462	14022.482	69572.006
NeEuropean	9931.110	6125.860	16282.749
NeEastAsian	7569.396	4673.461	11286.004
NeAndamanese	3858.272	2150.128	5637.242
NeIndian	8118.892	4894.395	11702.754
NePapuan	5076.282	3224.087	7626.228
NeAustralian	12309.795	6792.164	19259.710
NeNeanderthal	3918.538	1384.270	5875.804
NeDenisovan	2888.284	1353.423	3890.069
NeErectus	10943.903	1781.151	19414.566
*tSplitPapua_AA**	31.37	15.82	43.01
NePapua_AA	10141.002	2577.949	19173.600
*tsplitAndamese_India**	37.77	28.87	43.11
NeEAsia_India	15996.938	8755.218	19810.871
*tSplitAsia_Pacific**	47.6	43.77	51.88
NeAsia_Pacific	14614.393	7011.954	19762.659
*tSplitEuropean_Asia_Pacific**	56.60	52.33	67.89
NeOutOfAfrica	2786.436	1071.906	6105.232
*tSplitEurasia_Africa**	137.68	95.77	171.43
NeHumans	29728.932	13158.831	39292.484
NeBottleneckEurasia	3354.725	1209.499	4923.358
NeArchaics	14481.352	5792.521	23939.461
*tSplitNeanderthal_Denisova**	320.25	300.68	360.48
NeHominin	21400.065	13545.418	30645.232
*tHominin**	531.23	469.94	587.7
*tIntrogressionDenisovan_Pacific**	43.12	31.97	50.32
IntrogressionDenisovan_Pacific	0.016	0.004	0.025
*tIntrogressionErectus_Denisovan**	68.018	42.92	96.54
IntrogressionErectus_Denisovan	0.011	0.002	0.025
NeErectus_Hominin	46706.916	11924.078	92384.701

*tHominin_Erectus**	1474.61	1025.78	1972.40
NeUnknown	10409.164	1395.235	19748.086
*tIntrogressionAsia_Pacific**	53.73	45.75	64.68
IntrogressionUnknown_Asia_Pacific	0.034	0.019	0.048
*tIntrogressionNeanderthal_ModernHumanOutOfAfrica**	77.49	58.81	111.15
IntrogressionNeanderthal_Eurasia	0.019	0.007	0.034
NeDenisovanUnknown	13428.450	3881.706	19710.933
*tSplitDenisovan_Unknown**	279.15	206.25	359.88

* KYA assuming a generation time of 29 years.

Supplementary Table 10. Posterior distributions of the time of divergence between Neanderthal and Denisovan and between the archaic population source in each model and the unknown ghost archaic population (Xe).

**Model**	***tSplitNeanderthal_Denisova (95 CI)***	***tSplitXe (95 CI)***
**E**	312,37 (300,53 , 339,18)	261,3 (202,6 , 351,01)
**F**	320,25 (300,68 , 360,48)	279,1 (206,25 , 359,9)
**G**	310,75 (299,98 , 332,48)	416,9 (333,77 , 511,76)

Supplementary Figure 5. Posterior distributions (in black) obtained after running the ABC-DL approach with Model H. Red lines correspond to the prior distributions.

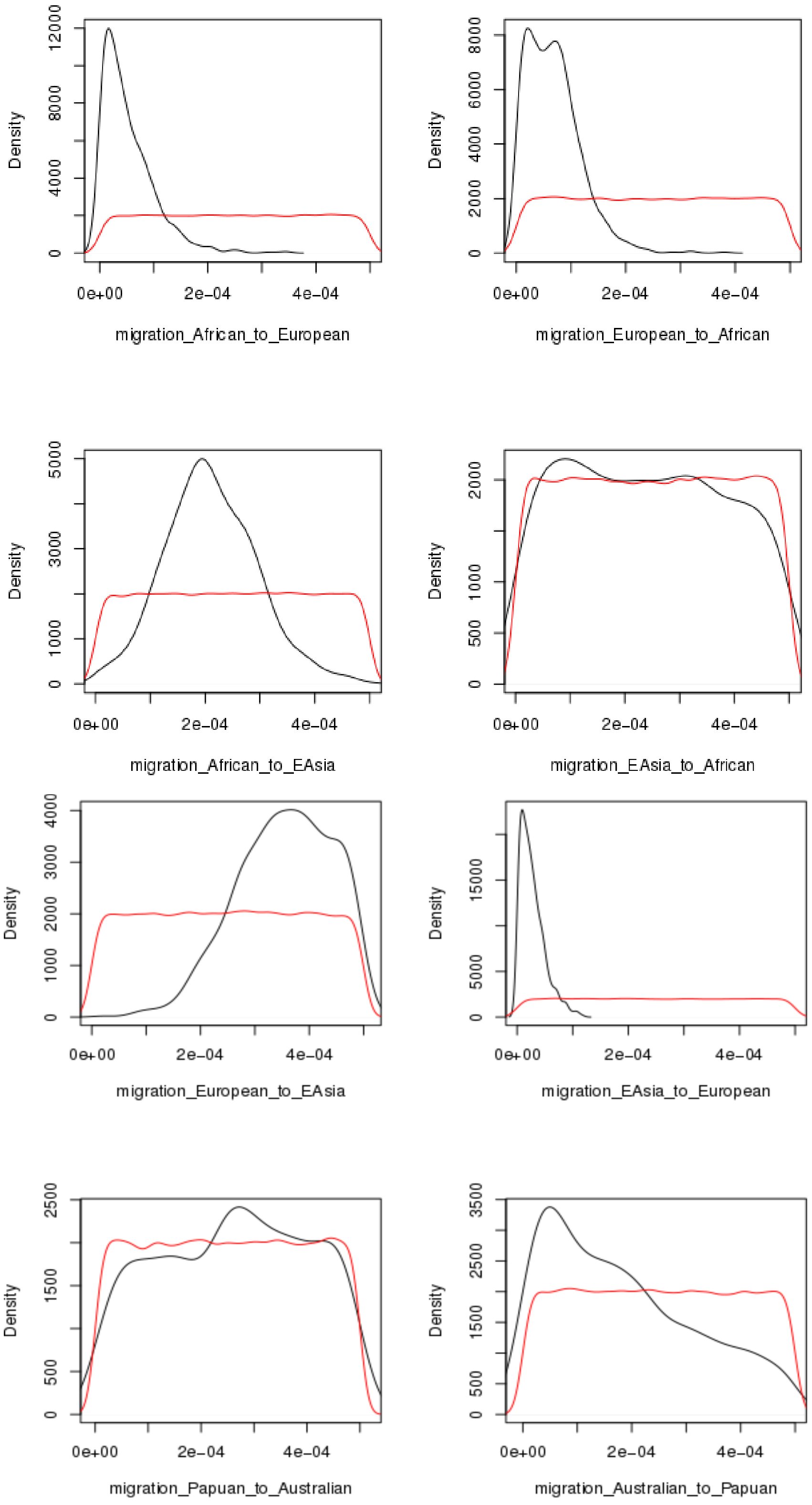

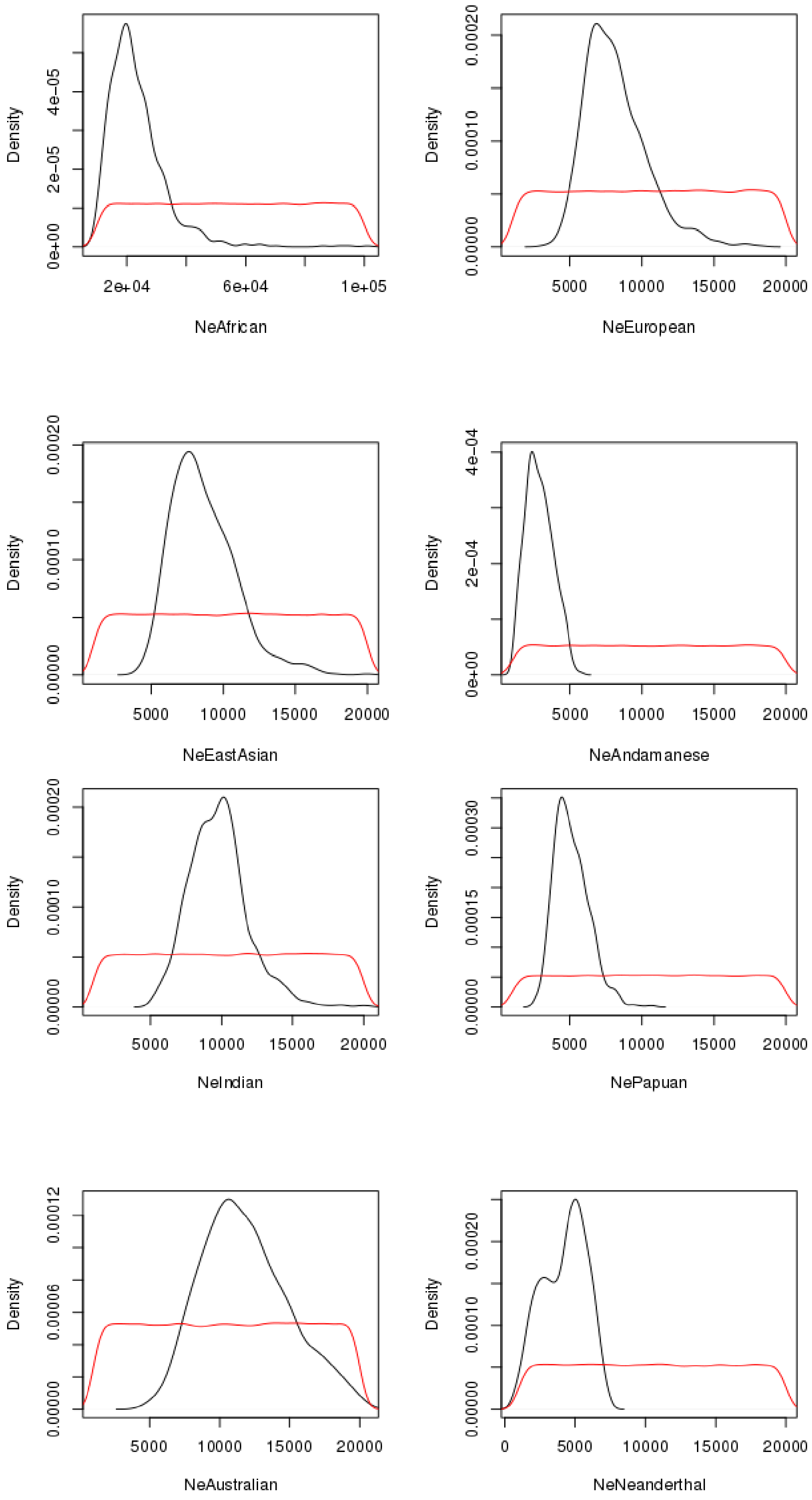
Density
0e+00
2e−05
4e−05
2e+04
6e+04
1e+05
NeAfrican
Density
0.00000
0.00010
0.00020
5000
10000
15000
20000
NeEuropean
Density
0.00000
0.00010
0.00020
5000
10000
15000
20000
NeEastAsian
Density
0e+00
2e−04
4e−04
5000
10000
15000
20000
NeAndamanese
Density
0.00000
0.00010
0.00020
5000
10000
15000
20000
NeIndian
Density
0.00000
0.00015
0.00030
5000
10000
15000
20000
NePapuan
Density
0.00000
0.00006
0.00012
5000
10000
15000
20000
NeAustralian
Density
0.00000
0.00010
0.00020
0
5000
10000
15000
20000
NeNeanderthal

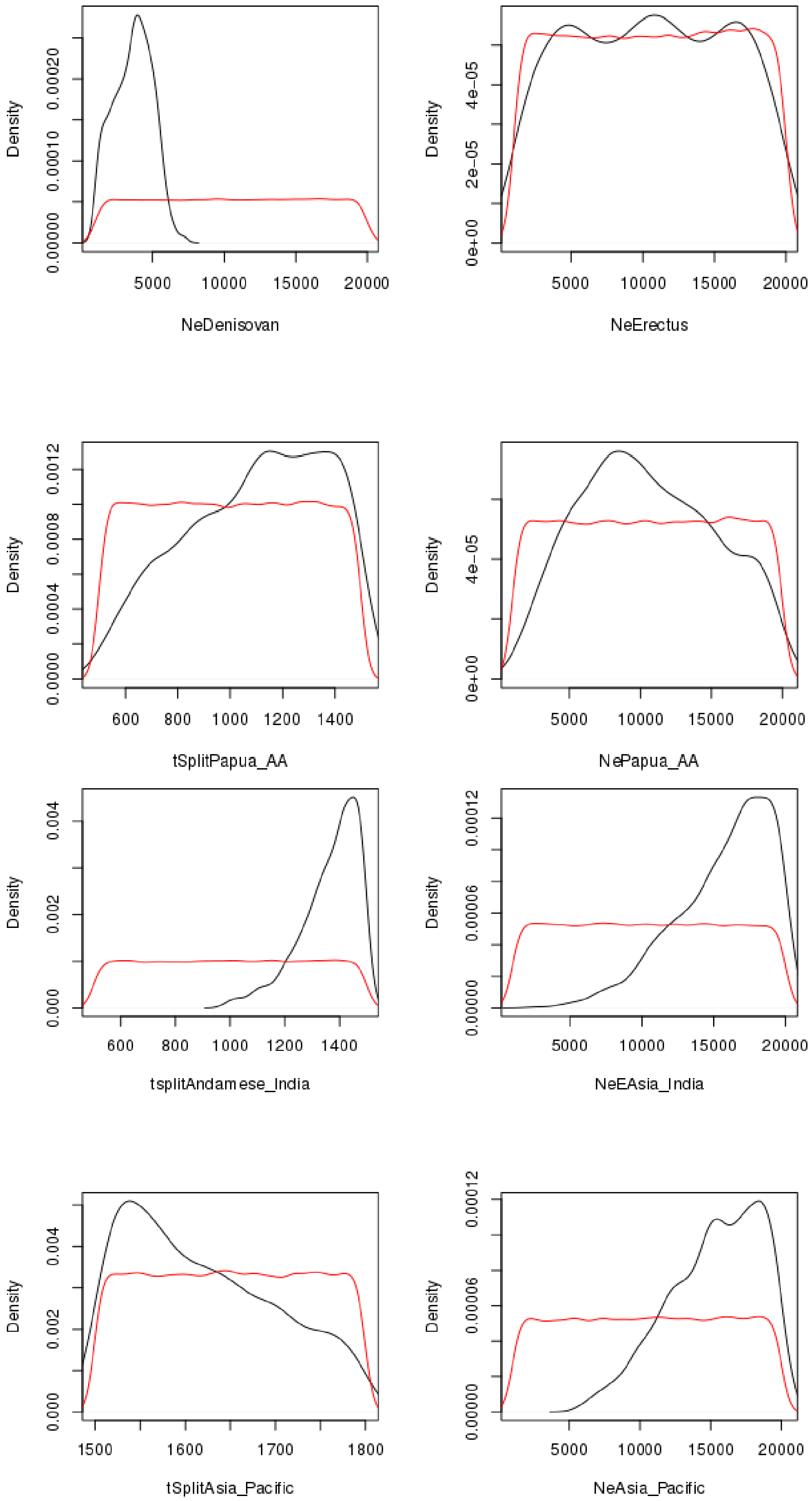
Density
0.00000
0.00010
0.00020
5000
10000
15000
20000
NeDenisovan
Density
0e+00
2e−05
4e−05
5000
10000
15000
20000
NeErectus
Density
0.0000
0.0004
0.0008
0.0012
600
800
1000
1200
1400
tSplitPapua_AA
Density
0e+00
4e−05
5000
10000
15000
20000
NePapua_AA
Density
0.000
0.002
0.004
600
800
1000
1200
1400
tsplitAndamese_India
Density
0.00000
0.00006
0.00012
5000
10000
15000
20000
NeEAsia_India
Density
0.000
0.002
0.004
1500
1600
1700
1800
tSplitAsia_Pacific
Density
0.00000
0.00006
0.00012
5000
10000
15000
20000
NeAsia_Pacific

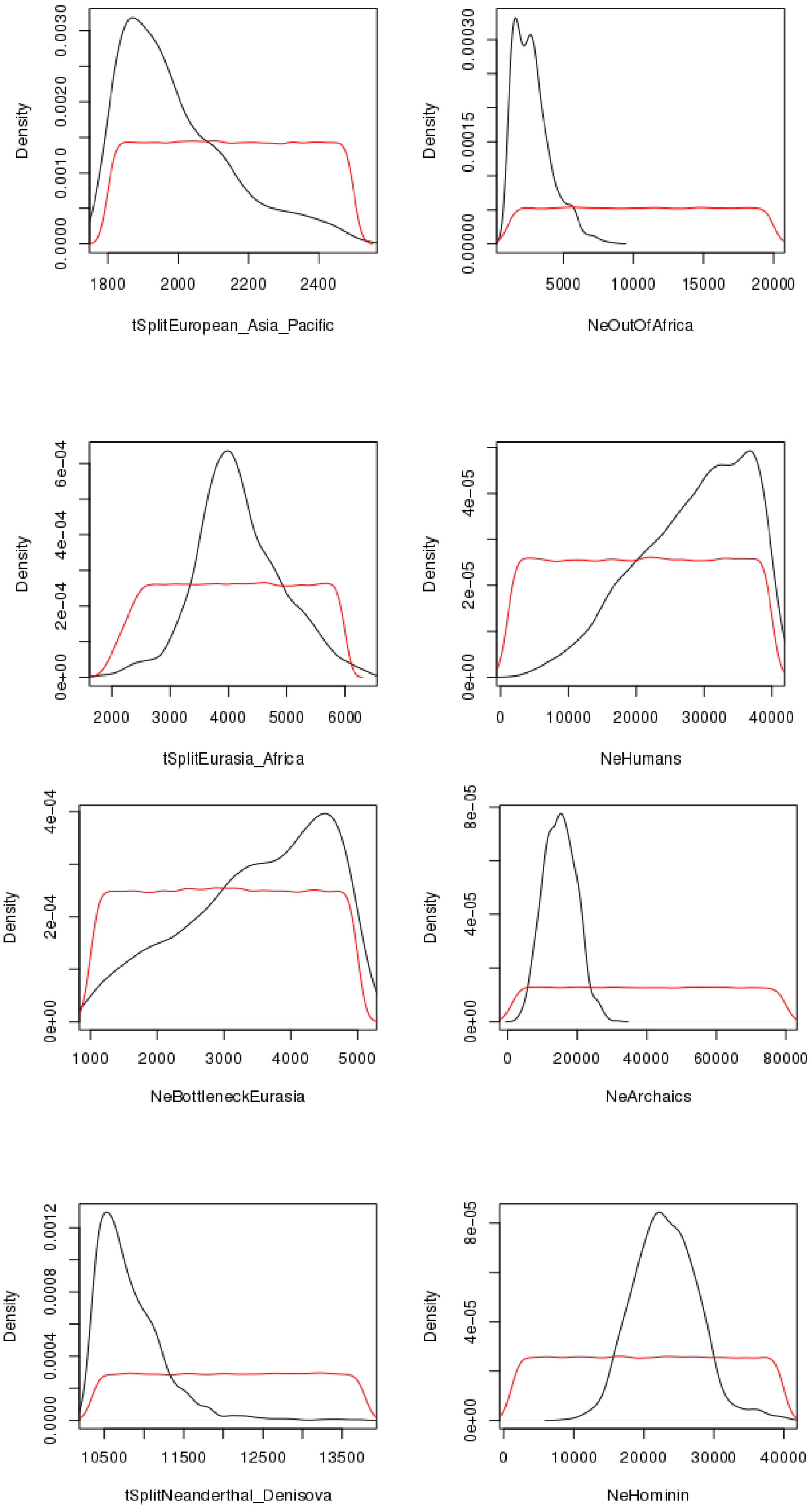
Density
0.0000
0.0010
0.0020
0.0030
1800
2000
2200
2400
tSplitEuropean_Asia_Pacific
Density
0.00000
0.00015
0.00030
5000
10000
15000
20000
NeOutOfAfrica
Density
0e+00
2e−04
4e−04
6e−04
2000
3000
4000
5000
6000
tSplitEurasia_Africa
Density
0e+00
2e−05
4e−05
0
10000
20000
30000
40000
NeHumans
Density
0e+00
2e−04
4e−04
1000
2000
3000
4000
5000
NeBottleneckEurasia
Density
0e+00
4e−05
8e−05
0
20000
40000
60000
80000
NeArchaics
Density
0.0000
0.0004
0.0008
0.0012
10500
11500
12500
13500
tSplitNeanderthal_Denisova
Density
0e+00
4e−05
8e−05
0
10000
20000
30000
40000
NeHominin

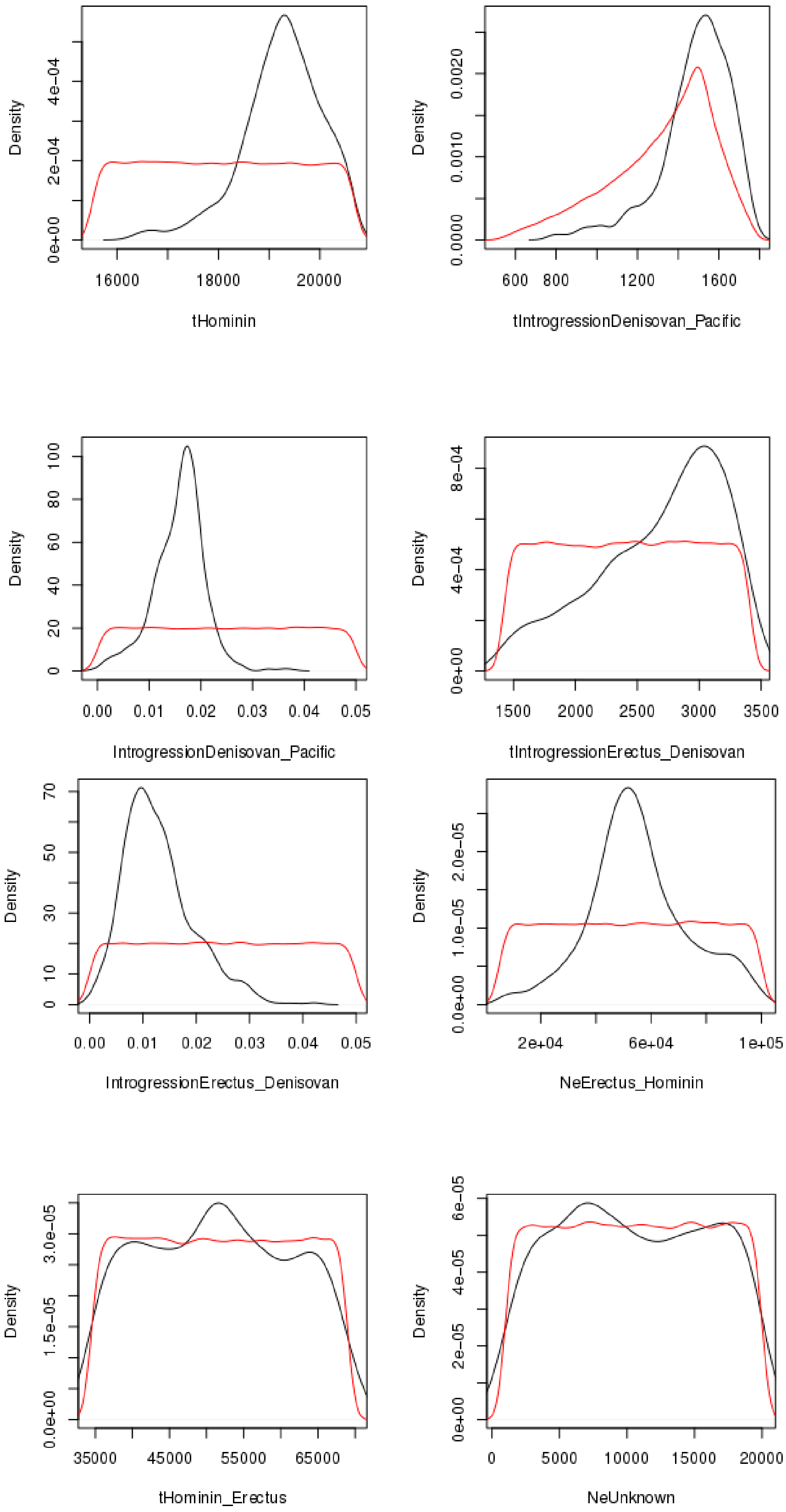
Density
0e+00
2e−04
4e−04
16000
18000
20000
tHominin
Density
0.0000
0.0010
0.0020
600
800
1200
1600
tIntrogressionDenisovan_Pacific
Density
0
20
40
60
80
100
0.00
0.01
0.02
0.03
0.04
0.05
IntrogressionDenisovan_Pacific
Density
0e+00
4e−04
8e−04
1500
2000
2500
3000
3500
tIntrogressionErectus_Denisovan
Density
0
10
30
50
70
0.00
0.01
0.02
0.03
0.04
0.05
IntrogressionErectus_Denisovan
Density
0.0e+00
1.0e−05
2.0e−05
2e+04
6e+04
1e+05
NeErectus_Hominin
Density
0.0e+00
1.5e−05
3.0e−05
35000
45000
55000
65000
tHominin_Erectus
Density
0e+00
2e−05
4e−05
6e−05
0
5000
10000
15000
20000
NeUnknown

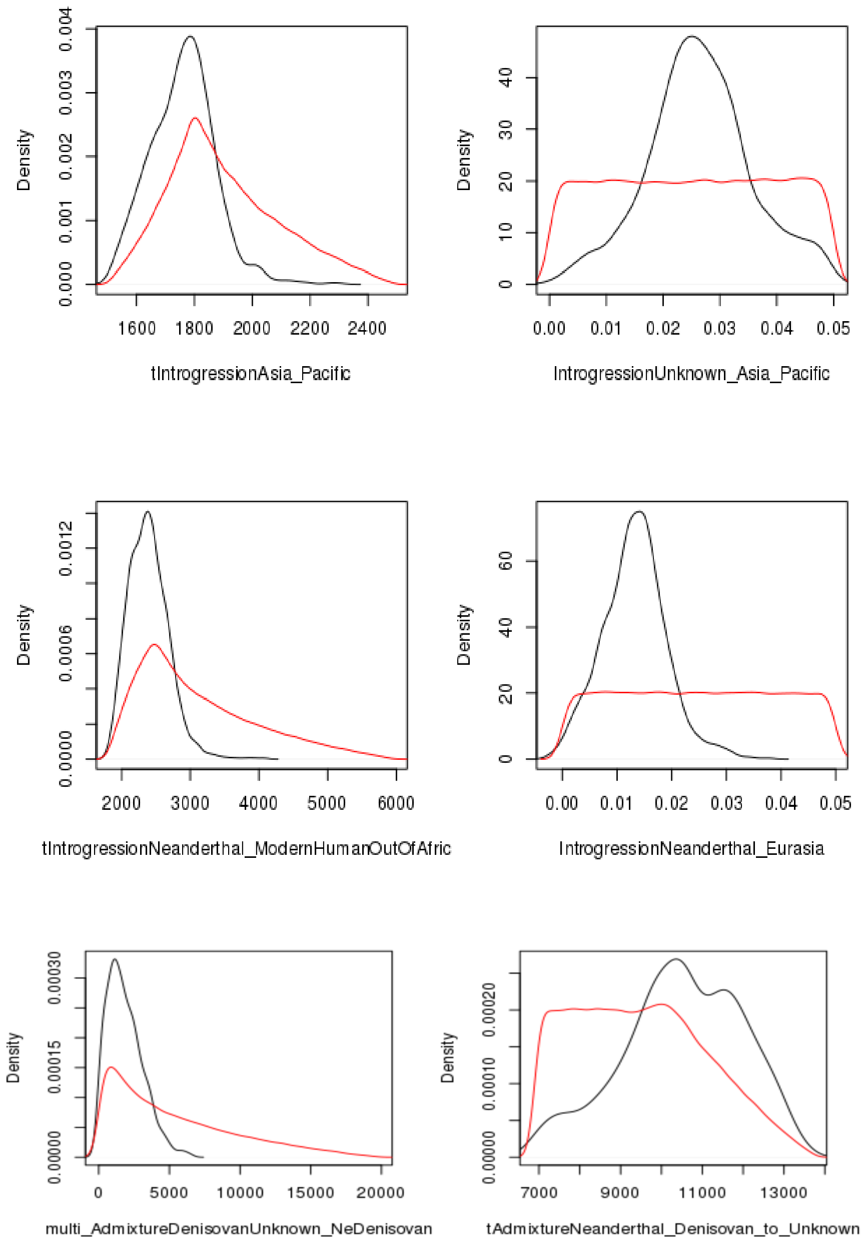
Density
tIntrogressionAsia_Pacific
IntrogressionUnknown_Asia_Pacific
tIntrogressionNeanderthal_ModernHumanOutOfAfric
IntrogressionNeanderthal_Eurasia
multi_AdmixtureDenisovanUnknown_NeDenisovan
tAdmixtureNeanderthal_Denisovan_to_Unknown

Supplementary References:

1. Li, H. Aligning sequence reads, clone sequences and assembly contigs with BWA-MEM. *arXiv Prepr. arXiv* **00,** 3 (2013).
2. Li, H. *et al.* The Sequence Alignment/Map format and SAMtools. *Bioinformatics* **25,** 2078–2079 (2009).
3. McKenna, A. *et al.* The genome analysis toolkit: A MapReduce framework for analyzing next-generation DNA sequencing data. *Genome Res.* **20,** 1297–1303 (2010).
4. Abecasis, G. R. *et al.* An integrated map of genetic variation from 1,092 human genomes. *Nature* **491,** 56–65 (2012).
5. Sherry, S. T. *et al.* dbSNP: the NCBI database of genetic variation. *Nucleic Acids Res.* **29,** 308–311 (2001).
6. Danecek, P. *et al.* The variant call format and VCFtools. *Bioinformatics* **27,** 2156–2158 (2011).
7. Purcell, S. *et al.* PLINK: a tool set for whole-genome association and population-based linkage analyses. *Am. J. Hum. Genet.* **81,** 559–575 (2007).
8. Mallick, S. *et al.* The Simons Genome Diversity Project: 300 genomes from 142 diverse populations. *Nature* **538,** 201–206 (2016).
9. Hudson, R. R. Generating samples under a Wright-Fisher neutral model of genetic variation. *Bioinformatics* **18,** 337–338 (2002).
10. Bock, C., Walter, J., Paulsen, M. & Lengauer, T. CpG island mapping by epigenome prediction. *PLoS Comput. Biol.* **3,** e110 (2007).
11. Prüfer, K. *et al.* The complete genome sequence of a Neanderthal from the Altai Mountains. *Nature* **505,** 43–49 (2014).
12. Lecun, Y., Bengio, Y. & Hinton, G. Deep learning. *Nature* **521,** 436–444 (2015).
13. Goodfellow, I., Bengio, Y. & Courville, A. *Deep learning*. (MIT Press, 2017).
14. Sheehan, S. & Song, Y. S. Deep Learning for Population Genetic Inference. *PLoS Comput. Biol.* **12,** (2016).
15. Sietsma, J. & Dow, R. J. F. Creating artificial neural networks that generalize. *Neural Networks* **4,** 67–79 (1991).
16. Elliott, D. L., Elliott, D. L. & Elliott, D. L. *A better Activation Function for Artificial Neural Networks*. (1993).

17. Heaton, J. *Programming Neural Network s with Encog 2 in Java*. *Journal of Chemical Information and Modeling* **53,** (2013).
18. Fenner, J. N. Cross-cultural estimation of the human generation interval for use in genetics-based population divergence studies. *Am. J. Phys. Anthropol.* **128,** 415–423 (2005).
