# Supplementary material for: Approximate Bayesian computation with deep learning supports a third archaic introgression in Asia and Oceania
Source: Nat Commun. 2019 Jan 16;10:246. doi: 10.1038/s41467-018-08089-7 (PMC6335398; doi:10.1038/s41467-018-08089-7)
Supplement: Supplementary file 3 — Reporting Summary [file 41467_2018_8089_MOESM3_ESM.pdf]

nature research

Corresponding author(s): OSCAR LAO
JAUME BERTRANPETIT

Reporting Summary

Nature Research wishes to improve the reproducibility of the work that we publish. This form provides structure for consistency and transparency in reporting. For further information on Nature Research policies, see Authors & Referees and the Editorial Policy Checklist.

Statistical parameters

When statistical analyses are reported, confirm that the following items are present in the relevant location (e.g. figure legend, table legend, main text, or Methods section).

n/a	Confirmed	
☒	☐	The exact sample size (*n*) for each experimental group/condition, given as a discrete number and unit of measurement
☒	☐	An indication of whether measurements were taken from distinct samples or whether the same sample was measured repeatedly
☐	☒	The statistical test(s) used AND whether they are one- or two-sided<br>*Only common tests should be described solely by name; describe more complex techniques in the Methods section.*
☒	☐	A description of all covariates tested
☒	☐	A description of any assumptions or corrections, such as tests of normality and adjustment for multiple comparisons
☐	☒	A full description of the statistics including central tendency (e.g. means) or other basic estimates (e.g. regression coefficient) AND variation (e.g. standard deviation) or associated estimates of uncertainty (e.g. confidence intervals)
☐	☒	For null hypothesis testing, the test statistic (e.g. *F*, *t*, *r*) with confidence intervals, effect sizes, degrees of freedom and *P* value noted<br>*Give P values as exact values whenever suitable.*
☐	☒	For Bayesian analysis, information on the choice of priors and Markov chain Monte Carlo settings
☒	☐	For hierarchical and complex designs, identification of the appropriate level for tests and full reporting of outcomes
☒	☐	Estimates of effect sizes (e.g. Cohen's *d*, Pearson's *r*), indicating how they were calculated
☐	☒	Clearly defined error bars<br>*State explicitly what error bars represent (e.g. SD, SE, CI)*

Our web collection on statistics for biologists may be useful.

Software and code

Policy information about availability of computer code

Data collection	No software was used for retrieving data. Raw data was directly obtained from different publicly available web repositories. FASTQ sequences were mapped using BWA mem 0.7.15 1 on GR37 reference genome using default parameters. BWA output (SAM format) was converted to binary BAM format using SAMtools 1.3.1 2 and sorted using picard tools 2.2.1 with SortSam using their coordinates. We realigned indels using IndelRealigner with already known indel interval file (1000 Genome Project 1st Phase Indel) 4 and recalibrated bases using BaseRecalibrator and PrintReads with dbSNP version 138 5 using GATK 3.5. BAM file was called for variants using HaplotypeCaller. VCF file was constructed with GenotypegVCFs. We removed all the SNPs which had missing information for any individual, using PLINK-1.07.
Data analysis	Admixtools1.4 was used to calculate the D-statistics.<br>ms software was used for simulating sequences.<br>FastSimcoal2 software was used for simulating sequences.<br>Encog 3.0 JAVA framework was used for conducting the neural networks.<br>R 3.5.0 software was used for conducting abc analyses, estimating summary statistics, etc and optimizing the models using D-statistics.<br>abc package in R software was used for conducting abc analyses<br>The pipeline to generate simulated data in the ABC-DL and computing the DL networks was generated in JAVA 1.8.<br>The pipeline is available at https://github.com/oscarlao/ABC_DL

For manuscripts utilizing custom algorithms or software that are central to the research but not yet described in published literature, software must be made available to editors/reviewers upon request. We strongly encourage code deposition in a community repository (e.g. GitHub). See the Nature Research guidelines for submitting code & software for further information.

Data

Policy information about availability of data

All manuscripts must include a data availability statement. This statement should provide the following information, where applicable:

- Accession codes, unique identifiers, or web links for publicly available datasets
- A list of figures that have associated raw data
- A description of any restrictions on data availability

All the sequence data came from their respective publicly available data sources and all other downstream data analysis are available on request to the corresponding authors.

Field-specific reporting

Please select the best fit for your research. If you are not sure, read the appropriate sections before making your selection.

☐ Life sciences ☐ Behavioural & social sciences ☒ Ecological, evolutionary & environmental sciences

For a reference copy of the document with all sections, see nature.com/authors/policies/ReportingSummary-flat.pdf

Ecological, evolutionary & environmental sciences study design

All studies must disclose on these points even when the disclosure is negative.

Study description	In this study we have developed a new approach based on combining Approximate Bayesian Computation with Deep Learning to make inferences about "ghost" archaic introgressions. We applied it to the study of Eurasian human populations.
Research sample	We used publicly available data. Full genomes were retrieved from 1000 genomes project, Simons Genome Diversity Panel, Indian populations from the paper of Mondal et al 2016, Altai, Vindija and Denisova archaic genomes. The data comprised samples from Sub-Saharan Africa, Europe, East Asia, India and Oceania, as well as the publicly available archaic genomes.
Sampling strategy	Since we were using the information from the whole sequenced genome, all our ABC analyses were based in one individual of each population used as noise injection during the training of the deep learning algorithm and one individual used for the ABC-DL inference. In the case of the archaic genomes, we used them both in the training and replication. Performance of the algorithm based on this sample size were tested by means of simulated data.
Data collection	We retrieved the data from databases publicly available in internet. We downloaded raw FASTQ data and inferred the genotypes using different software.
Timing and spatial scale	All data was retrieved at once. This parameter was not relevant for our analyses.
Data exclusions	We did not exclude data. This data is already curated by their original owners.
Reproducibility	For D-stats and F4 ratio, we conducted a bootstrapping in order to estimate the standard error of the estimates.<br>For model comparison in ABC-DL, we computed a confusion matrix using simulated data of known models and estimating by means of ABC-DL the most supported model.<br>For parameter estimation, we estimated the performance of the DL by computing in a replication dataset the Pearson correlation between the value of the parameter used for the simulation and the predicted by the DL after the training.
Randomization	This is not really relevant for our study, since we were conducting analyses to estimate the topology and demographic parameters of demographic models using the assigned individuals to their respective populations.
Blinding	Blinding was not required in our analyses since we were considering population data in which the label of the individual was required for conducting the analyses.

Did the study involve field work? ☐ Yes ☒ No

Reporting for specific materials, systems and methods

Materials & experimental systems

n/a	Involved in the study
☒	☐ Unique biological materials
☒	☐ Antibodies
☒	☐ Eukaryotic cell lines
☒	☐ Palaeontology
☒	☐ Animals and other organisms
☒	☐ Human research participants

Methods

n/a	Involved in the study
☒	☐ ChIP-seq
☒	☐ Flow cytometry
☒	☐ MRI-based neuroimaging
